# Computed tomography findings of pulmonary histoplasmosis: pictorial essay

**DOI:** 10.1590/0100-3984.2022.0106-en

**Published:** 2023

**Authors:** Ana Luiza Di Mango, Antônio Carlos Portugal Gomes, Bruno Hochhegger, Gláucia Zanetti, Edson Marchiori

**Affiliations:** 1 Universidade Federal do Rio de Janeiro (UFRJ), Rio de Janeiro, RJ, Brazil; 2 Medimagem/BP Medicina Diagnóstica, São Paulo, SP, Brazil; 3 University of Florida, Gainesville, FL, USA

**Keywords:** Histoplasmosis, Mycoses, Tomography, X-ray computed, Histoplasmose, Micoses, Tomografia computadorizada

## Abstract

Endemic systemic mycoses are prevalent in specific geographic areas of the world and are responsible for high rates of morbidity and mortality in the populations of such areas, as well as in immigrants and travelers returning from endemic regions. Pulmonary histoplasmosis is an infection caused by *Histoplasma capsulatum*, a dimorphic fungus. This infection has a worldwide distribution, being endemic in Brazil. Histoplasmosis can affect the lungs, and its diagnosis and management remain challenging, especially in non-endemic areas. Therefore, recognition of the various radiological manifestations of pulmonary histoplasmosis, together with the clinical and epidemiological history of the patient, is essential to narrowing the differential diagnosis. This essay discusses the main computed tomography findings of pulmonary histoplasmosis.

## Abstract (pt)

As micoses sistêmicas endêmicas são prevalentes em áreas geográficas específicas do mundo e são responsáveis por altas taxas de morbidade e mortalidade nessas populações e em imigrantes e viajantes que retornam de regiões endêmicas. A histoplasmose pulmonar é uma infecção causada pelo *Histoplasma capsulatum*, um fungo dimórfico. Essa infecção tem distribuição mundial, apresentando-se de forma endêmica no Brasil. A histoplasmose pode afetar os pulmões de pacientes, e seu diagnóstico e manejo permanecem desafiadores, especialmente em áreas não endêmicas. Portanto, o reconhecimento das várias manifestações radiológicas da histoplasmose pulmonar associadas a história clínica e epidemiológica dos pacientes é fundamental para estreitar o diagnóstico diferencial. Este ensaio discute os principais achados tomográficos da histoplasmose pulmonar.

## INTRODUCTION

Pulmonary histoplasmosis is an infection caused by *Histoplasma capsulatum*, a dimorphic fungus. This infection has a worldwide distribution and is the most common endemic pulmonary mycosis in the United States, as well as in Central and South America. There are also reports of its occurrence in Africa, Asia (including China and India), and (rarely) in Europe. Histoplasmosis is endemic in Brazil. The fungus can be isolated from places where the soil is contaminated with droppings from birds or (especially) bats, such as caves, mines, old buildings, hollow trees, and chicken coops. Activities such as landscaping, demolition of old buildings, and cleaning of attics and barns, as well as soil tilling, are associated with exposure and dissemination of the infectious particles.

Pulmonary histoplasmosis is characterized by nonspecific clinical manifestations and has a wide spectrum of clinical signs and symptoms, its presentations ranging from asymptomatic to severe and fatal. It can also present in acute, subacute, and chronic forms. In immunocompetent individuals, the acute form usually presents as subclinical or self-limited illness. In the subacute form, the pulmonary involvement is mild but persistent and can last for weeks or months. The chronic form is uncommon and is seen mainly in patients with structural lung disease, such as chronic obstructive pulmonary disease. Disseminated histoplasmosis is typically seen in patients with advanced HIV infection (those with a CD4 count below 150 cells/mL) or who are otherwise immunocompromised^(1-6)^.

The gold standard for the diagnosis of histoplasmosis is the direct identification of *H. capsulatum* in tissues or body fluids, with or without its isolation in culture. Noninvasive diagnostic methods include testing for antigens (in urine or serum) and for antibodies (through complement fixation or immunodiffusion). Antibody testing may be negative in immunosuppressed patients. The utility of polymerase chain reaction testing remains unclear^(2,3,6)^.

The imaging findings of pulmonary histoplasmosis are varied and nonspecific. Therefore, it is essential to take the epidemiological history of the patient in order to support the diagnostic suspicion. Computed tomography (CT) is the imaging method of choice for evaluating such patients. The most common CT patterns in pulmonary histoplasmosis are nodular opacities (solitary or multiple), consolidations, and ground-glass opacities. It should be borne in mind that pulmonary histoplasmosis is the fungal disease that most closely mimics neoplastic lung disease. In endemic areas, the finding of a solitary pulmonary nodule should raise the suspicion of pulmonary histoplasmosis. The use of positron-emission tomography/CT (PET/CT) is of little use in these cases, because infectious/inflammatory diseases and malignant lesions are usually hypermetabolic, with high levels of FDG uptake and consequent false-positive results for neoplasia^(7-10)^.

In view of the high prevalence of pulmonary histoplasmosis, it is necessary to be familiar with the clinical manifestations, epidemiological aspects, and CT manifestations, early diagnosis and appropriate treatment being essential to slow the progression of the disease. In this pictorial essay, we review and illustrate the common and uncommon presentations of thoracic histoplasmosis.

## TOMOGRAPHIC MANIFESTATIONS

The CT findings of pulmonary histoplasmosis are varied and nonspecific. Acute pulmonary histoplasmosis is usually self-limited, and radiological examinations demonstrate ill-defined, diffuse, bilateral pulmonary opacities, with or without mediastinal or hilar lymph node enlargement. In the subacute form, those opacities tend to be more focal and typically resolve spontaneously, in some cases evolving to calcified pulmonary nodules or calcified mediastinal lymph nodes. Patients with the acute form of the disease, notably those with some degree of immunosuppression, can present with extrapulmonary dissemination, manifesting as pericarditis, hepatosplenomegaly, skin alterations, and rheumatologic disorders^(7-10)^.

### Nodules

**Figure 1:**
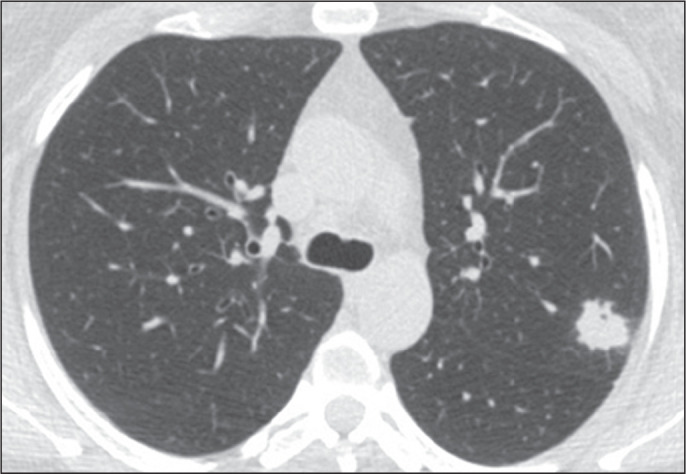
CT of an asymptomatic 36-year-old woman who tested positive for histoplasmosis on immunodiffusion, showing a nodule, with soft-tissue density and irregular, spiculated contours, in the upper left lobe.

**Figure 2:**
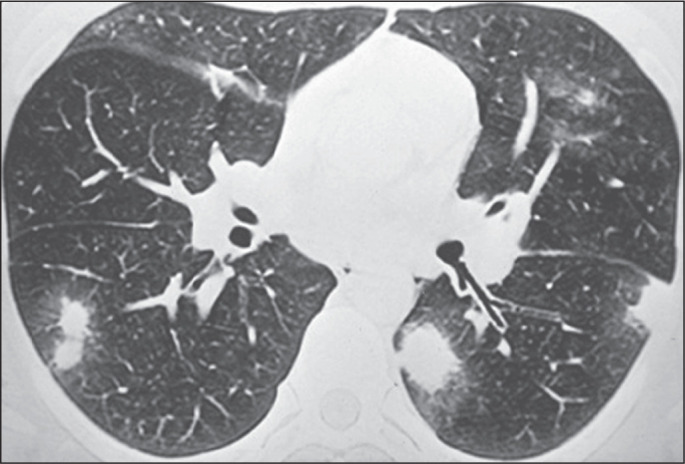
CT of a 47-year-old woman, showing multiple nodules of varying sizes with ground-glass halos in both lungs.

**Figure 3:**
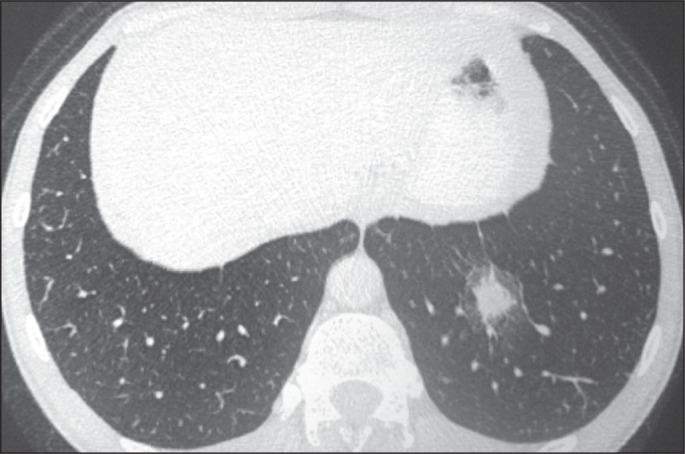
CT of a 47-year-old man with positive serology, showing a nodule surrounded by ground-glass attenuation (the halo sign) in the left lower lobe.

**Figure 4:**
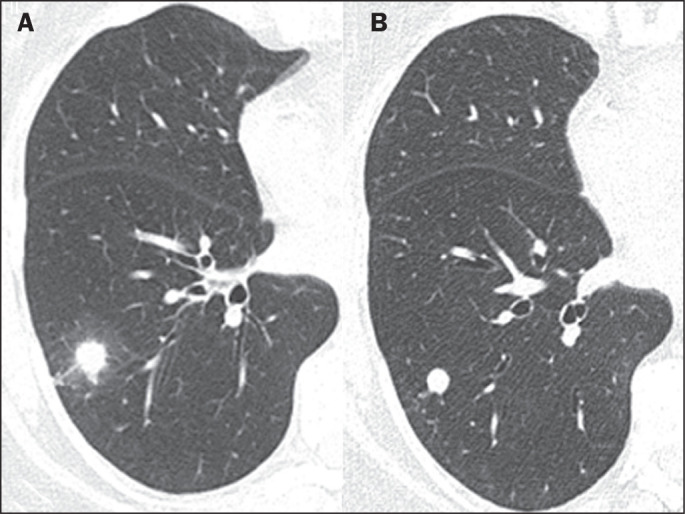
A 57-year-old man. A: CT showing a nodule with a ground-glass halo in the right lower lobe. B: Control CT examination performed nine months later, showing a nodule with smooth, regular contours, without a ground-glass halo.

**Figure 5:**
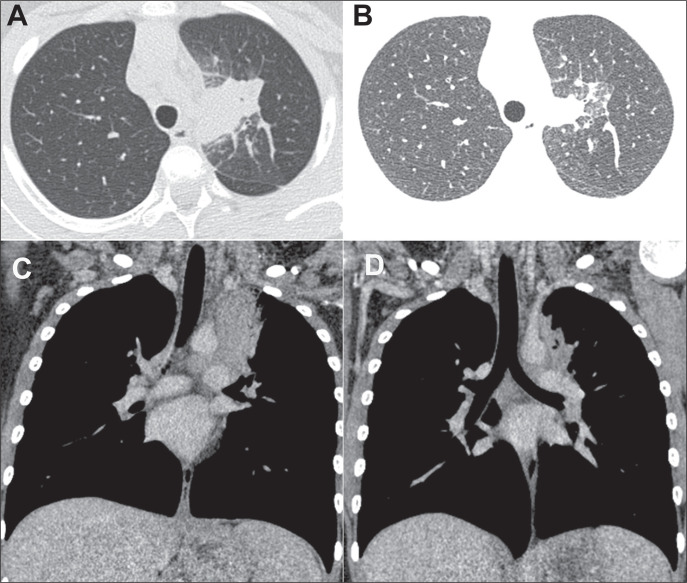
A 9-year-old girl. A,B: CT scans showing a mass in the upper left lobe, adjacent to the mediastinum, with irregular borders, initially interpreted as a neoplasm and testing positive for histoplasmosis on immunodiffusion. C,D: Control CT scans acquired one month later, showing significant regression of the lesion.

**Figure 6:**
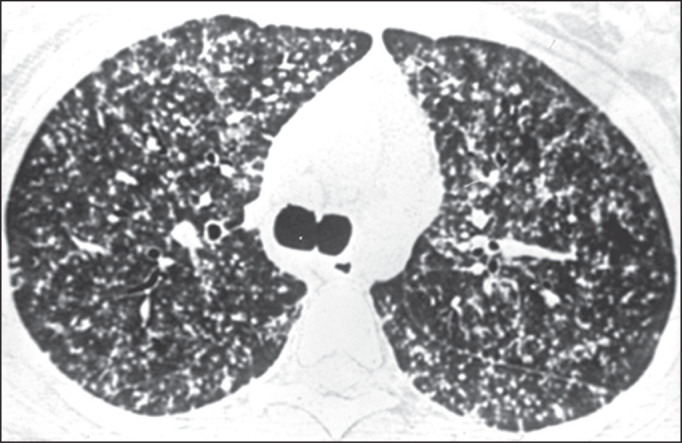
CT of a 39-year-old man, showing small pulmonary nodules with a random, diffuse distribution, mimicking miliary tuberculosis.

**Figure 7:**
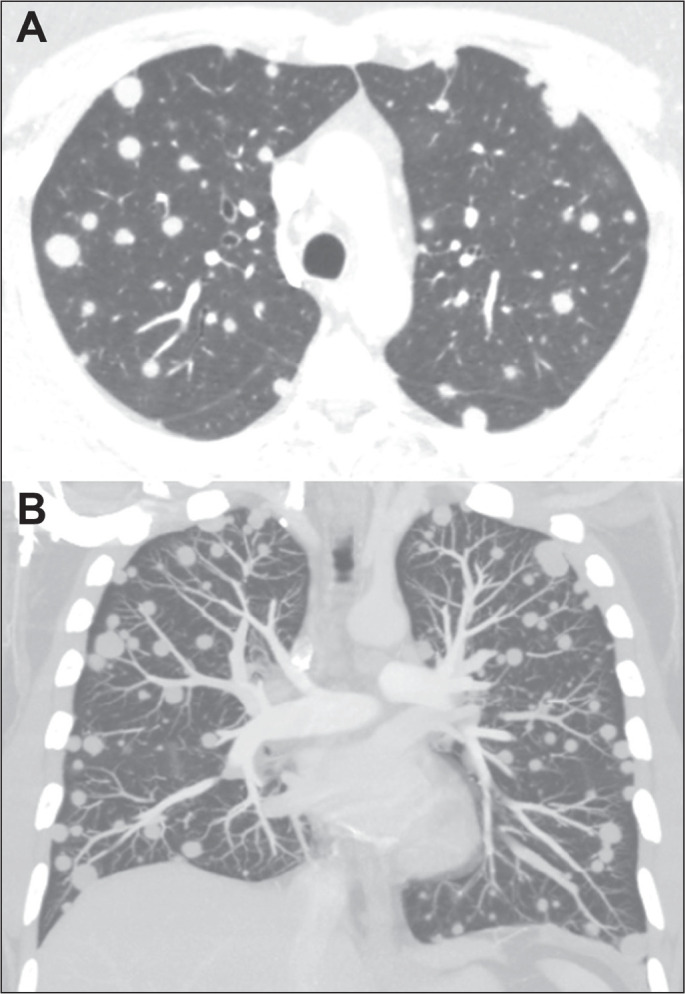
CT of a 40-year-old man diagnosed with pulmonary histoplasmosis by nodule biopsy, showing multiple nodules of varying sizes, disseminated in both lungs, mimicking hematogenous metastases.

Pulmonary nodules, either solitary (Figure 1) or multiple (Figure 2), are the most common findings in pulmonary histoplasmosis, occurring mainly in residents of endemic areas. Such nodules are typically asymptomatic. As illustrated in Figures 3 and 4, they can vary in size and can have smooth or irregular contours, with or without a ground-glass halo (the halo sign). They can also show central necrosis or calcifications. In some cases, they have the appearance of a mass (Figure 5). Cavitation is rare in such nodules. In some cases, they present a miliary pattern of distribution (Figure 6), miliary tuberculosis being the main differential diagnosis. They can also mimic hematogenous metastases (Figure 7). On imaging examinations, a solitary pulmonary nodule can mimic a malignant lung lesion.

### Histoplasmoma

**Figure 8:**
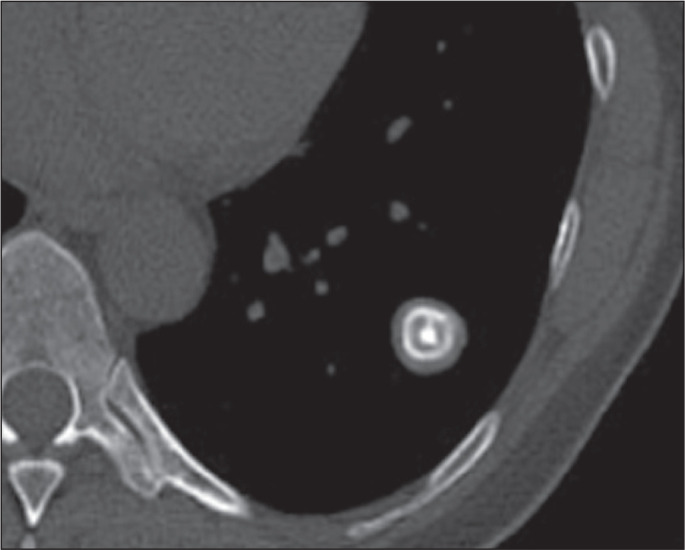
CT of a 37-year-old man with a histoplasmoma, showing a nodule, with smooth contours and concentric laminar calcifications, in the left lower lobe.

Histoplasmomas are pulmonary nodules that are typically solitary, with or without calcifications. When present, the calcifications can have a central, diffuse, or laminar pattern. The laminar pattern of calcification (Figure 8), although nonspecific, is highly suggestive of a histoplasmoma. Histologically, a histoplasmoma is characterized by foci of necrosis surrounded by fibrotic tissue around a previously formed granuloma. Most patients with histoplasmoma are asymptomatic. Therefore, when a solitary pulmonary nodule is found in an asymptomatic patient, the hypothesis of histoplasmoma should be considered, especially in known endemic regions.

### Consolidation

**Figure 9:**
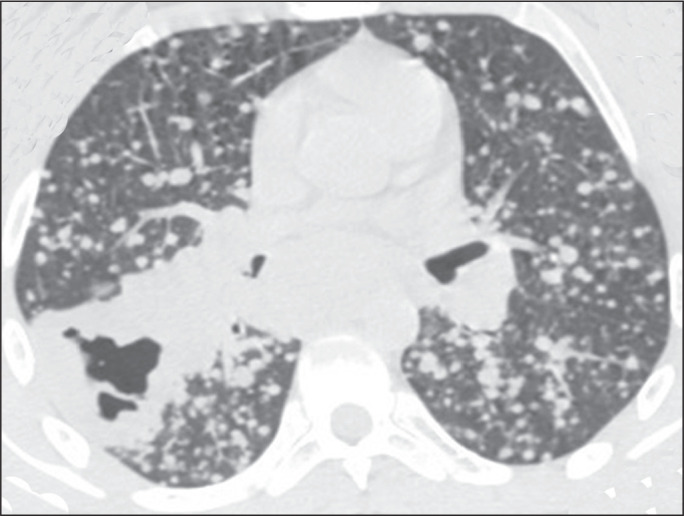
CT of a 29-year-old man, showing a cavitary mass, with internal septation, in the right lower lobe, together with multiple nodules disseminated throughout the lungs. Note also the lymph node mass in the subcarinal region.

Clinically, acute histoplasmosis can present as an influenza-like illness or community-acquired bacterial pneumonia, with cough, fever, chest discomfort, myalgia, and headache. On CT, acute pulmonary histoplasmosis can manifest as irregular consolidation involving one or several lobes, mimicking bacterial pneumonia, organizing pneumonia, or neoplasia. Those consolidations can show cavitation (Figure 9). Concomitant hilar and mediastinal lymph node enlargement is common.

### Chronic cavitary pulmonary histoplasmosis

Chronic cavitary lung disease is an uncommon manifestation of histoplasmosis. It is seen almost exclusively in men with chronic obstructive pulmonary disease. Typically, the initial imaging manifestation of chronic histoplasmosis is a segmental area of consolidation. Like tuberculosis, chronic pulmonary histoplasmosis typically involves the apical and posterior segments of the upper lobes. A CT scan shows chronic consolidation with progressive cavitation, resulting in volume loss, in the upper lobe. Pleural thickening adjacent to apical cavitary lesions is common. The cavitations can evolve to affect the entire lung lobe, completely destroying it and reducing its volume.

### Disseminated histoplasmosis

**Figure 10:**
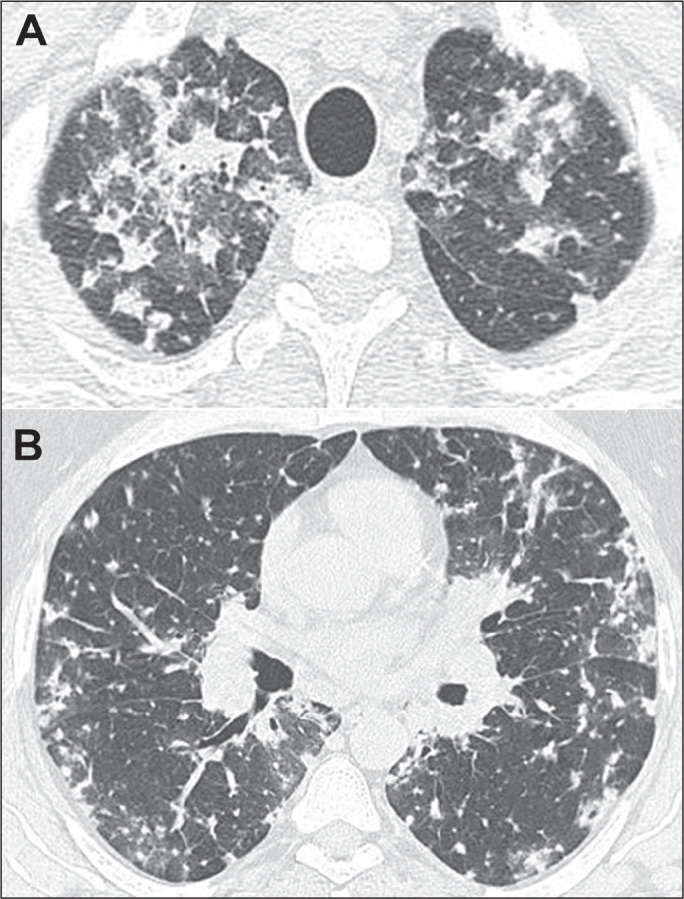
CT of a 57-year-old man with positive serology, showing disseminated histoplasmosis. Note the focal areas of consolidation and ground-glass attenuation, together with small nodules, throughout both lungs.

Disseminated histoplasmosis typically occurs in immunocompromised patients. In severe cases, it can present as sepsis with hypotension, disseminated intravascular coagulation, renal failure, or acute respiratory distress. On imaging, the most common pulmonary manifestation of disseminated histoplasmosis is diffuse pulmonary micronodules, which can be misdiagnosed as miliary tuberculosis or hematogenous metastases. Disseminated histoplasmosis can also present as airspace opacities, which can be segmental, lobar, or diffuse (Figure 10).

### Broncholithiasis

**Figure 11:**
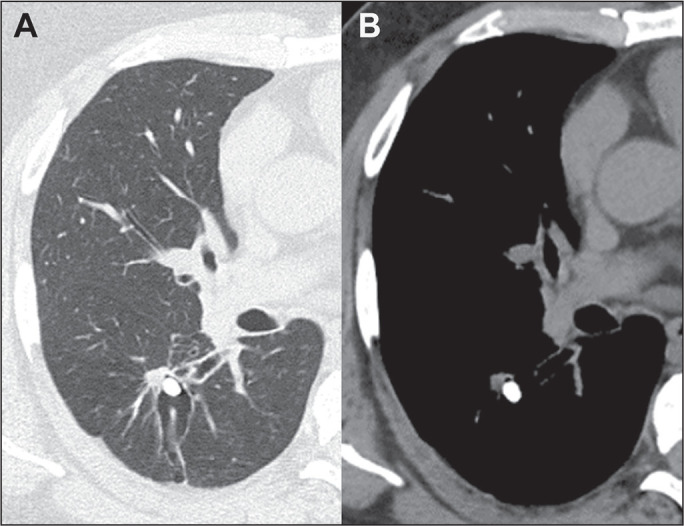
CT of a 51-year-old man with cough and dyspnea, diagnosed with broncholithiasis. Note the bronchiectasis, encompassing a small calcified nodule, in the right lower lobe.

Broncholithiasis is a late, uncommon pulmonary complication of histoplasmosis. It occurs when a calcified peribronchial nodule or calcified mediastinal or hilar lymph node erodes into the airway. On chest CT, it manifests as a calcification occluding the bronchus and results in atelectasis of the lung lobe distal to the obstruction (Figure 11).

### Fibrosing mediastinitis

**Figure 12:**
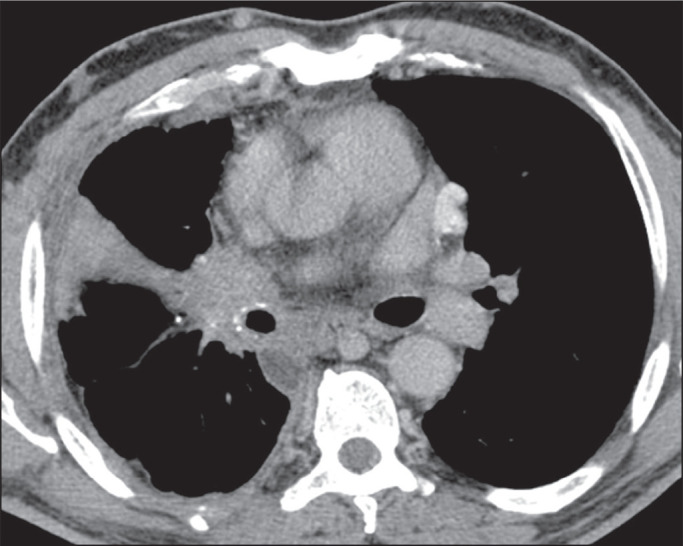
CT of a 51-year-old woman with fibrosing mediastinitis. Histoplasmosis was not identified on the biopsy but was diagnosed by immunodiffusion. Note the infiltrative lesion in the subcarinal and hilar lymph nodes on the right, containing foci of calcification, as well as the atelectasis in the middle lobe.

Among the various complications of pulmonary histoplasmosis, fibrosing mediastinitis is the most severe, with the highest morbidity and mortality. It is characterized by excessive proliferation of fibrotic tissue around the mediastinal lymph nodes. This fibrotic proliferation can lead to incarceration of mediastinal structures, the most common finding being obstruction of the superior vena cava. The airways, pulmonary circulation, and esophagus can also be affected. Chest CT reveals an infiltrative mass with soft-tissue density obliterating the mediastinal adipose layers and incarcerating adjacent structures, typically containing extensive calcifications (Figure 12).

### Lymph node enlargement

**Figure 13:**
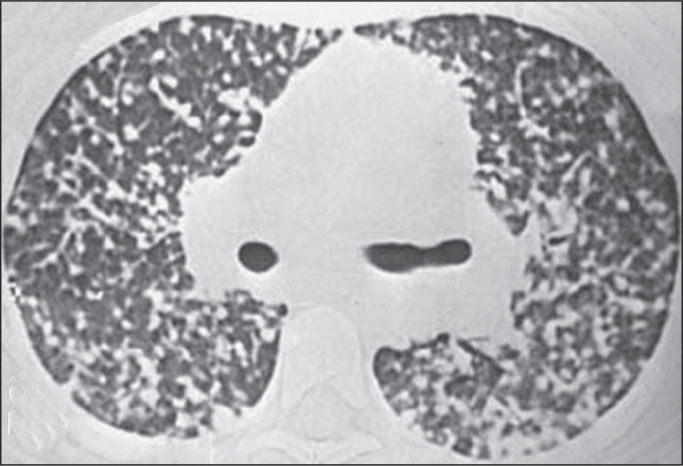
CT of a 6-year-old with positive serology, showing small nodules disseminated throughout both lungs. Note also the bilateral hilar lymph node enlargement.

Acute pulmonary histoplasmosis can result in mediastinal and hilar lymph node enlargement, which can be voluminous and exert a mass effect on the adjacent structures (Figure 13). Lymph nodes may show FDG uptake on ^18^F-FDG PET/CT, simulating malignant disease. As they heal, infected mediastinal lymph nodes may calcify.

## CONCLUSION

In the acute and chronic forms of pulmonary histoplasmosis, there is a wide spectrum of thoracic manifestations. On imaging examinations, histoplasmosis can be indistinguishable from other infectious diseases, inflammatory conditions, and neoplasms. A diagnostic hypothesis of pulmonary histoplasmosis should be considered in patients residing in or coming from endemic areas, and radiologists should be familiar with its various imaging manifestations.
